# Building on Sub-Arctic Soil: Geopolymerization of Muskeg to a Densified Load-Bearing Composite

**DOI:** 10.1038/s41598-017-15115-z

**Published:** 2017-11-07

**Authors:** Gregory R. Waetzig, Junsang Cho, Max Lacroix, Sarbajit Banerjee

**Affiliations:** 1 Department of Chemistry, Texas A&M University, College Station, TX 77843-3255 USA; 2 Department of Materials Science & Engineering, Texas A&M University, College Station, TX 77843 USA; 3 Cenovus Energy, Inc., 500 Centre St. S., Calgary, AB T2P 0M5 Canada

## Abstract

The marshy water-saturated soil typical of the sub-Arctic represents a considerable impediment to the construction of roads, thereby greatly hindering human habitation and geological excavation. Muskeg, the native water-laden topsoil characteristic of the North American sub-Arctic, represents a particularly vexing challenge for road construction. Muskeg must either be entirely excavated, or for direct construction on muskeg, a mix of partial excavation and gradual compaction with the strategic placement of filling materials must be performed. Here, we demonstrate a novel and entirely reversible geopolymerization method for reinforcing muskeg with wood fibers derived from native vegetation with the addition of inorganic silicate precursors and without the addition of extraneous metal precursors. A continuous siloxane network is formed that links together the muskeg, wood fibers, and added silicates yielding a load-bearing and low-subsidence composite. The geopolymerization approach developed here, based on catalyzed formation of a siloxane network with further incorporation of cellulose, allows for an increase of density as well as compressive strength while reducing the compressibility of the composite.

## Introduction

Access to fossil fuel deposits in the Canadian oil sands and in the sub-Arctic is greatly hindered by the difficulties of constructing roads or drilling pads on vast areas of muskeg, the native organic marshy topsoil saturated with water^1–5^. The ready subsidence of muskeg limits earthworks operations to the Arctic winter when the ground is frozen to solid ice or alternatively creates considerable safety and technical challenges when operations are conducted in warmer temperatures. Muskeg comprises mosses, sedges, grasses, and remnants of plant matter decomposed to various extents. In order to facilitate construction, the muskeg must either be entirely excavated and replaced with a compacted engineered fill, or for direct construction on muskeg, a mix of partial excavation and gradual compaction with the strategic placement of filling materials must be performed to ensure an acceptable degree of settlement^4,6–8^. The former approach is prohibitively expensive and labor-intensive, whereas the latter approach may require many months of settlement. The muskeg layers are generally frozen ca. 0.3 m below the ground surface during winter. Techniques have been developed to increase the ice depth to provide safe access to remote areas, but thicker ice layers limit settlement control, which is key to avoiding muskeg failure. Layers can be reinforced with biaxial geogrids or geosynthetics but these necessitate expensive capital investments and are still subject to inhomogeneous settling^4,6,8^. In contrast, the subgrade presents a stiff soil that is more amenable to construction. Upon conditioning, the native low-to-medium plastic clay and clay till can also be excavated and used to backfill excavated regions or placed atop the muskeg layer. However, this again is labor intensive and requires considerable excavation; ensuring fills remain unfrozen during compaction also adds an additional level of complexity making winter earthworks a considerable challenge. Consequently, finding routes to build directly on muskeg has emerged as an urgent imperative for ensuring the viability of sub-Arctic fossil fuel deposits.

Geopolymerization involves the construction of silicate and aluminosilcate solid matrices, typically for purposes such as the sequestration of toxic sludge^9,10^, and provides a potential route for solidification of muskeg. Geopolymers can be cast to specific dimensions, formed into load-bearing structural elements, or deployed within concrete roadways. An attractive potential solution to the challenges of building on muskeg involves the infiltration of liquid precursors within the muskeg layer followed by cross-linking to constitute a solid densified load-bearing matrix. However, such solidification and hardening of the muskeg matrix has thus far not been achievable given the ca. 900% moisture content of muskeg and environmental requirements that limit the incorporation of aluminum and transition-metal frameworks given the relatively low natural abundance of these elements in native muskeg. In this work, we demonstrate the transformation of muskeg to a solid composite based on the formation of inorganic silicate frameworks that are further reinforced by incorporation of locally sourced cellulosic material derived from mulch. An unprecedented densified load-bearing composite matrix is obtained with no extraneous metal contamination, suggesting an entirely new approach for all-weather earthworks in marshy sub-Arctic environments and substantially mitigating the need for excavation and importation of engineered geofills. The structural framework thus constituted can furthermore be readily dissolved, allowing for restoration of the natural habitat.

## Results and Discussion

### Formation of Reinforcing Silicate Framework within Muskeg

**Figure 1:**
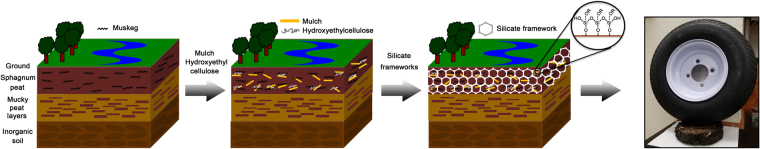
Illustration of geopolymerization approach developed to solidify muskeg to a load-bearing silicate composite. Schematic depiction of strengthening and solidification of muskeg based on infiltration of aqueous precursors for formation of a siloxane network further reinforced by the addition of mulch fibers and hydroxyethylcellulose.

Strengthening and reinforcing muskeg soils through aqueous solidification chemistry^11^ holds great promise for facilitating direct construction on muskeg by forming a load-bearing cross-linked covalently bonded matrix. Figure 1 illustrates the modification and strengthening of muskeg based on infiltration of silica precursors that are catalytically cross-linked to constitute an amorphous siloxane framework. The network is reinforced with naturally occurring wood fibers; hydroxyethylcellulose is further added to promote additional cross-linking by dint of its hydroxyl groups. Free-standing pucks of muskeg with dimensions ranging up to 19.0 cm in diameter and 7.0 cm in height are prepared at 25 °C by mixing wet muskeg soil with naturally occurring wood-fiber mulch and hydroxyethylcellulose, followed by addition of the silicate precursor and initiation of acid- or base-catalyzed condensation. Elimination of free water and solidification occurs within 3–5 days^12–14^. Comparable muskeg specimens have also been prepared at a constant temperature of −25 °C in order to mimic the sub-Arctic environment. Lower temperatures retard the kinetics of framework formation but solidified frameworks are obtained within 7–10 days. The wood fibers are derived from mulch that has the same geographical origin as the muskeg; the hydroxyethylcellulose further provides a means to covalently bond the wood fibers to the incipient silica framework. The use of these naturally sourced materials to facilitate solidification instead of commonly used metal-based geopolymer additives minimizes the environmental impact of the proposed densification process^15,16^. Indeed, the network can be readily dissolved by base treatment suggesting a facile means of restoring the soil to its native condition after use (*vide infra*). The formation of the siloxane framework within the muskeg matrix and the subsequent additional cross-linking induced by reaction with hydroxyethylcellulose results in expulsion of excess water and yields dry cross-linked free-standing samples.

The degree of cross-linking of the silicate frameworks is strongly affected by the nature of the silica precursors, the reaction pH, and the catalyst used. Indeed, the reaction of silicate precursors with cellulose nanocrystal templates has been previously reported by MacLachlan and co-workers in a different context^17–19^. These researchers have constructed chiral silica matrices by the templated cross-linking of sol—gel precursors in architectures defined by chiral cellulosic templates followed by calcination to remove the soft template; the specific chirality and texturation of the deposited silica depend sensitively on the pH, water content, and evaporation rate^19^. Analogously, these parameters are found to strongly influence the reaction of silica precursors within the composite soil matrices depicted in Fig. 1 and thereby strongly influence the extent of strengthening (or densification) of the muskeg soils^20–22^. In particular, the hydrolysis and condensation reaction kinetics are strongly pH-dependent, and this parameter determines the overall densification achieved. Two distinct silica precursors, Na_2_SiO_3_ and tetraethylorthosilicate (TEOS), have been examined under acidic and basic conditions (Supplementary Table 1). Supplementary Figure 1 contrasts the composite materials obtained for the two precursors under acidic and basic conditions. Na_2_SiO_3_ clearly yields more homogeneous samples with a uniform hue indicative of uniform infiltration, whereas white powdery regions are discernible upon use of TEOS as the precursor suggesting some phase segregation. Density measurements have further been used as a metric to contrast the extent of silica incorporation and densification. Table 1 lists the density values measured for composites prepared using Na_2_SiO_3_ as the precursor with varying amounts of mulch and hydroxyethylcellulose additives. Unmodified muskeg has a density of 0.2 g·cm^−3^, which is strongly enhanced upon infiltration of the silica precursors and formation of the siloxane framework reinforced with various amounts of mulch and hydroxyethylcellulose (0.6–1.05 g·cm;^−3^ Table 1). In contrast, the density values of comparable composites prepared using TEOS as the precursor are in the range of ca. 0.2–0.5 g·cm^−3^. For instance, when 10 mL of the silica precursor is used with 2 g of hydroxyethylcellulose and without the addition of supporting fillers, the Na_2_SiO_3_ precursor yields a density of 0.78 g·cm^−3^, whereas TEOS yields a density of 0.43 g·cm^−3^. The increased densification observed with the silicate precursor is attributed to the accelerated kinetics of hydrolysis/condensation reactions with this precursor as previously noted in the literature^23,24^. Sai *et al*. have examined the kinetics of the gelation process of TEOS and Na_2_SiO_3_ and have found that at room temperature the latter precursor is completely reacted within 2 s, whereas the TEOS precursor requires several minutes to a few hours^24,25^. In TEOS, the four ethoxide groups attached to the Si center have to be initially hydrolyzed by water or hydroxide (under acidic or basic conditions, respectively) to generate orthosilicic acid Si(OH)_4_ for further condensation reactions as per:

1 $${\rm{Si}}{({\rm{OR}})}_{{\rm{4}}}(l)+{{\rm{H}}}_{{\rm{2}}}{\rm{O}}(l)\to {\rm{Si}}{({\rm{OH}})}_{{\rm{4}}}(aq.)+{\rm{4ROH}}(aq.)$$

2 $${\rm{Si}}{({\rm{OH}})}_{{\rm{4}}}(aq.)+{\rm{Si}}{({\rm{OH}})}_{{\rm{4}}}(aq.)\mathop{\to }\limits^{N{H}_{4}OH}\,{({\rm{OH}})}_{{\rm{3}}}{\rm{Si}}\mbox{--}{\rm{O}}\mbox{--}{\rm{Si}}{({\rm{OH}})}_{{\rm{3}}}({\rm{s}})+{{\rm{H}}}_{{\rm{2}}}{\rm{O}}(l)$$

**Table 1:** Densification of Modified Muskeg Composites as a Function of Composition. Measured density of muskeg samples modified using a fixed amount of 10 g of wet muskeg with different concentrations of added mulch and hydroxyethylcellulose. Na_2_SiO_3_ is used as the precursor and the hydrolysis and condensation reaction is base catalyzed in each instance listed here.

Amount of Na_2_SiO_3_ (mL)	Amount of Mulch (g)	Amount of Catalyst (mL)	Amount of Additives (g)	Density (g/cm^3^)
Unmodified				0.20 ± 0.01 g/cm^3^
30 mL	0 g	10 mL	2 g	0.95 ± 0.08 g/cm^3^
30 mL	0 g	10 mL	4 g	0.73 ± 0.07 g/cm^3^
20 mL	5 g	10 mL	2 g	0.56 ± 0.05 g/cm^3^
30 mL	5 g	10 mL	2 g	1.02 ± 0.09 g/cm^3^
30 mL	5 g	10 mL	4 g	1.05 ± 0.08 g/cm^3^
20 mL	10 g	10 mL	1 g	0.62 ± 0.05 g/cm^3^
20 mL	10 g	5 mL	2 g	0.66 ± 0.06 g/cm^3^
10 mL	10 g	10 mL	2 g	0.55 ± 0.05 g/cm^3^
20 mL	10 g	15 mL	2 g	0.69 ± 0.06 g/cm^3^
20 mL	10 g	10 mL	2 g	0.61 ± 0.06 g/cm^3^
30 mL	10 g	10 mL	2 g	0.72 ± 0.07 g/cm^3^
20 mL	10 g	10 mL	3 g	0.70 ± 0.07 g/cm^3^
20 mL	15 g	10 mL	2 g	0.65 ± 0.06 g/cm^3^
30 mL	15 g	10 mL	2 g	0.76 ± 0.06 g/cm^3^
30 mL	15 g	10 mL	4 g	0.73 ± 0.06 g/cm^3^

In contrast, Na_2_SiO_3_ yields ionized corner-sharing oligomeric tetrahedral networks [SiO_3_]_*n*_^2−^ in water, as per:

3 $$n\cdot {{\rm{Na}}}_{2}{{\rm{SiO}}}_{3}\,(s)\to 2n\cdot {{\rm{Na}}}^{+}\,(aq.)+{[{{\rm{SiO}}}_{3}]}_{n}^{2-}\,(aq.)$$

The ready availability of oligomeric [SiO_3_]_*n*_^2−^ (where *n* is the degree of oligomerization) facilitates rapid formation of the siloxane backbone within the muskeg matrix, favors cross-linking with hydroxyl moieties on mulch and hydroxyethylcellulose (facilitating the incorporation of these additives within the monolithic composite), and yields an overall higher degree of condensation of silicate frameworks in comparison to TEOS where monomeric Si(OH)_4_ need to be individually connected as per Eq. 2. In terms of equilibrium considerations, given the high water content in the muskeg matrix, the formation of a siloxane network as per Eq. 2 is hindered due to the competing back reaction. Given the significantly improved results with Na_2_SiO_3_, only this precursor is further examined for subsequent optimization of reaction conditions.

**Figure 2:**
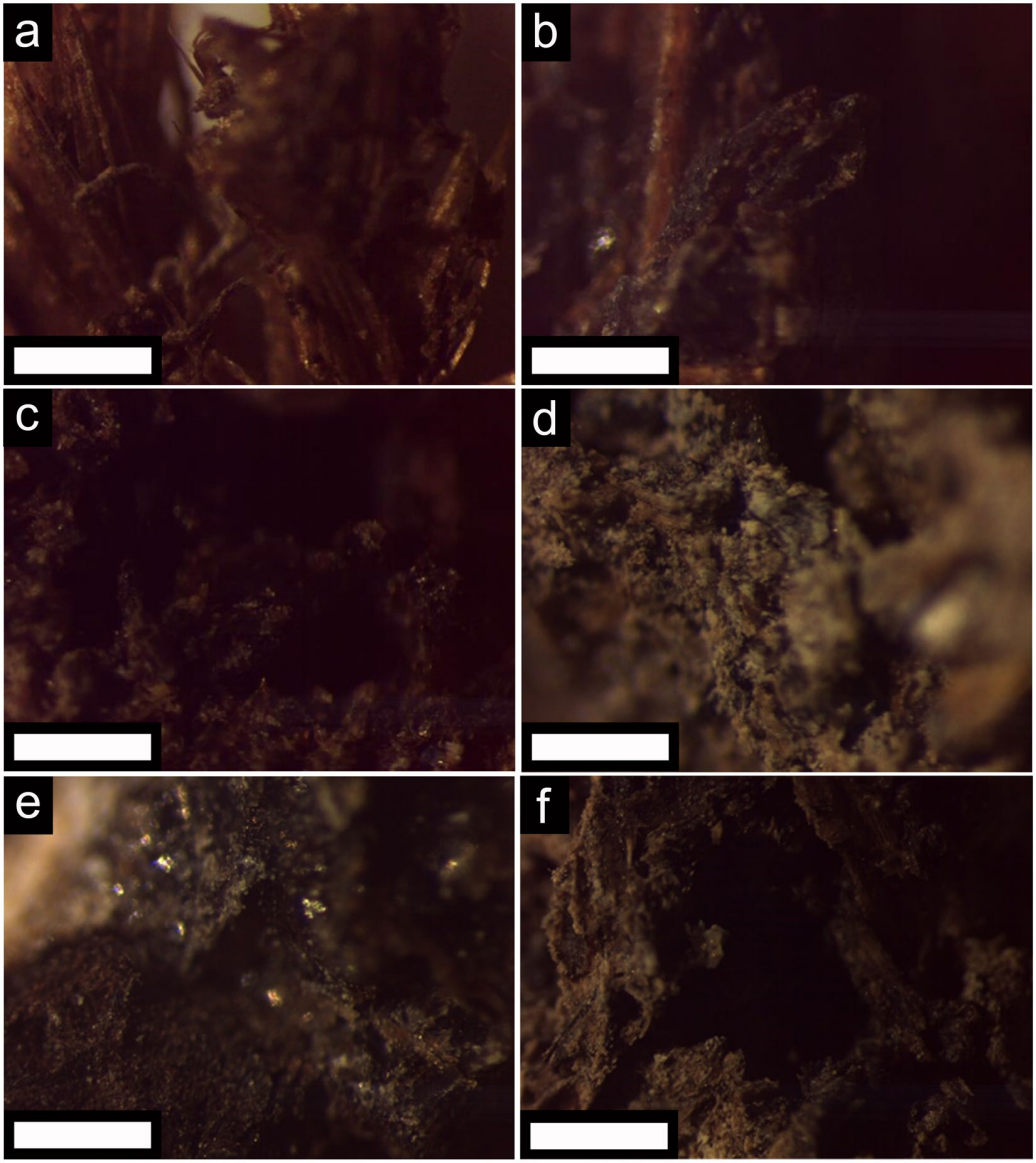
Optical Microscopy Imaging of Muskeg and Reinforced Composites. Stereomicroscopy images of (**a**) unmodified and (**b**‒**f**) modified muskeg with different concentrations of added Na_2_SiO_3_ and mulch: (**b**) 20 mL and 10 g; (**c**) 20 mL and 5 g; (**d**) 20 mL and 15 g; (**e**) 30 mL and 5 g; and (**f**) 30 mL and 15 g. The amount of NH_4_OH and hydroxyethylcellulose were held constant for each modified muskeg specimen at 10 mL and 2 g, respectively. Scale bar = 1 mm.

Muskeg itself is a mildly acidic soil comprising dead plants, peat soils, and excess water^1–3^. The color, gross morphological features, and structure of dried muskeg layers have been evaluated by stereomicroscopy as indicated in Fig. 2. Muskeg drained of water by straining comprises dark brown fibrous matter, predominantly disintegrated cellulosic materials^2^. Upon infiltration of Na_2_SiO_3_, hydrolysis, and condensation, the composite remains homogeneous but a shiny more reflective hue reflecting the formation of siloxane layers is discernible across the entire matrix.

**Figure 3:**
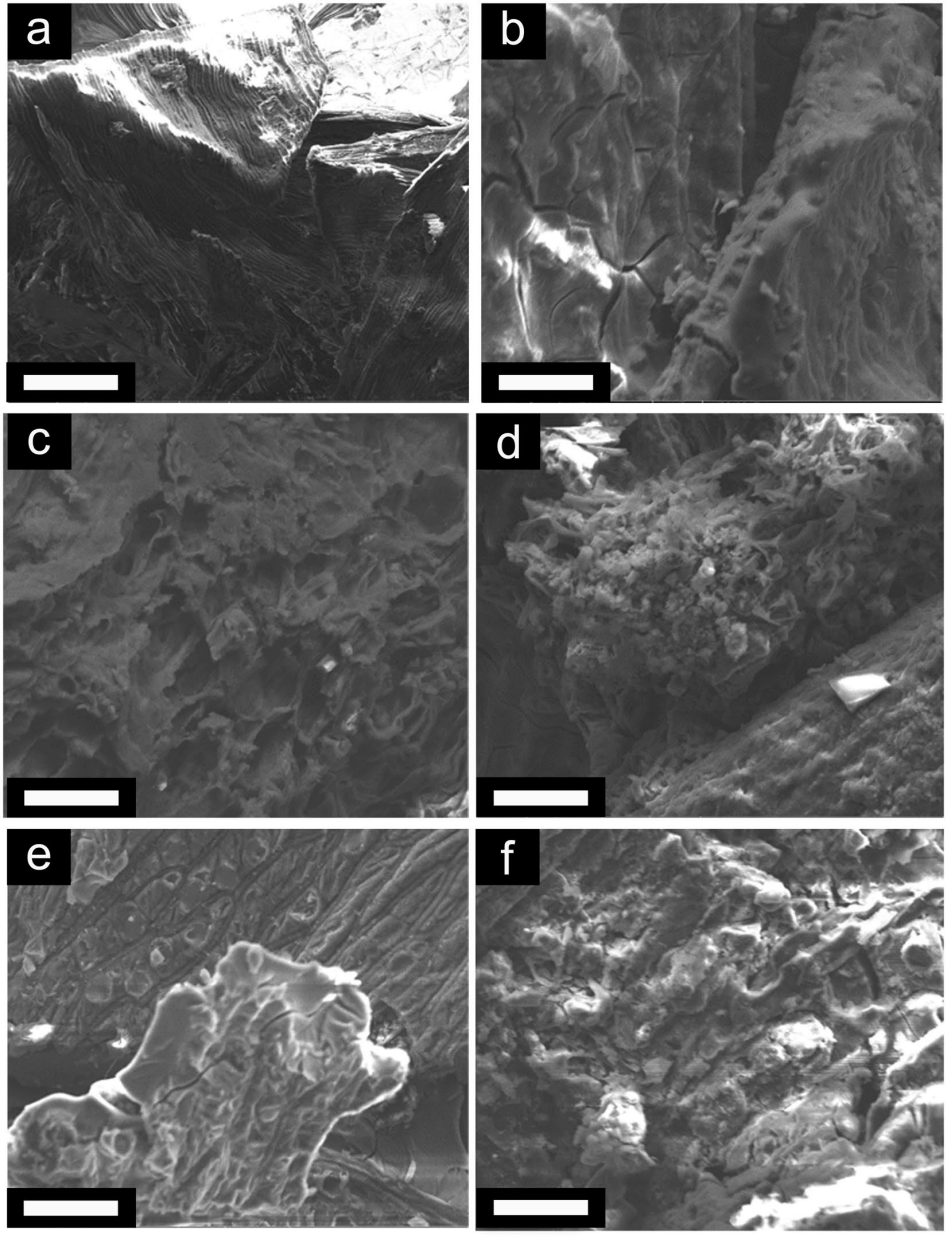
Electron Microscopy Characterization of Reinforced Muskeg. SEM images of (**a**) unmodified muskeg and (**b**‒**f**) modified muskeg with different added ratios of sodium silicate and mulch: (**b**) 20 mL and 10 g; (**c**) 20 mL and 5 g; (**d**) 20 mL and 15 g; (**e**) 30 mL and 5 g; and (**f**) 30 mL and 15 g. The hydroxyethylcellulose and NH_4_OH content was held constant for each modified muskeg specimen at 2 g and 10 mL, respectively. Scale bar = 50 μm.

The influence of pH on the hydrolysis/condensation reactions and chemical stability of modified muskeg has further been evaluated^26^. The muskeg soils themselves are slightly acidic with a pH of 6.82. The pH value is decreased to 2.13 when using TEOS and HCl catalyst whereas an increase in pH to 8.40 occurred when using TEOS and NH_4_OH catalyst. The pH values for Na_2_SiO_3_ as the precursor are 0.37 when using HCl as the catalyst and 10.94 when using NH_4_OH as the catalyst (Supplementary Table 1). The fundamental mechanism of the dissociative reaction (hydrolysis) of Na_2_SiO_3_ precursors is the nucleophilic attack of water (under acidic conditions) or hydroxide anions (under basic conditions) on the silicon centers. Under acidic conditions, protonated silicic acid is more susceptible to the nucleophilic attack of water molecules owing to the inductive effect, whereas under basic conditions, nucleophilic substitution is favored based on a direct nucleophilic attack of hydroxide ions^27^. However, when exposed to either strongly acidic or basic conditions, the reverse condensation reaction (depolymerization) is facilitated since water molecules created as a result of the gelation process can, in the presence of a catalyst, nucleophilically attack and cleave the Si-O-Si bonds (reverse reaction of Eq. 2). Consequently, upon using HCl as a catalyst, the low pH conditions (pH < 2.5) result in fragmentation of the composite frameworks within less than 24 h. Indeed, monolithic free-standing specimens could not be stabilized for either of the precursors, Na_2_SiO_3_ or TEOS, under highly acidic conditions (Supplementary Figure 1)^26,28^. It is likely that the low pH also damages the cellulosic fibers derived from the wood mulch that form a network within the composite matrix compromising their ability to hold together the framework. In contrast, modified muskeg prepared under basic conditions (pH < 11) using NH_4_OH as the catalyst are weight bearing and form homogenous densified specimens as depicted by the digital photographs in the insets to Fig. 3.

### Characterization of Reinforced Muskeg

The morphological characteristics of modified muskeg prepared using Na_2_SiO_3_ as the precursor and NH_4_OH as the catalyst have been investigated by scanning electron microscopy (SEM) (Fig. 3). As observed in stereomicroscopy images (Fig. 2), unmodified muskeg exhibits a fibrous cellular structure. The constituent fibers are aligned and assembled within aggregates upon dehydration (Fig. 2a). However, upon infiltration and condensation of Na_2_SiO_3_, this fibrous morphology is no longer observed and instead amorphous silica deposition is observed completely enveloping the muskeg layers within continuous films (Fig. 2b and c) that appear to conformally coat the fibrous aggregates; the films are characterized by micro-cracks originating from removal of water (Fig. 2b)^29^. Supplementary Figure 2 shows the presence of sub-micron-sized aggregates of silica particles at the surfaces of the modified muskeg. While there is some interesting literature precedence on the ability of semi-crystalline cellulosic materials to induce some degree of crystallinity in templated continuous polymeric matrices^30,31^, Supplementary Figure 3 indicates that the templated silica is entirely amorphous upon deposition onto the mulch fibers. With increasing relative content of added Na_2_SiO_3_, a higher density of silica particles are observed at the surfaces (Fig. 3e,f) corresponding to homogeneous nucleation of silica in addition to the conformal heterogeneously nucleated siloxane network coating the muskeg. The chemical composition of muskeg samples before and after assembly of the siloxane framework has been further analyzed by energy dispersive X-ray spectroscopy (EDS, Supplementary Figure 4). The unmodified muskeg contains carbon (ca. 69.3 at.%), oxygen (ca. 29.7 at.%), and some aluminum (1.1 at.%, of mineral origin). The infiltration and condensation of silica precursors results in the appearance of sodium (4.1‒8.1 at.%) and silicon (4.8‒8.1 at.%), depending on the amount of Na_2_SiO_3_ used. EDS mapping suggests a homogeneous distribution of Si, C, and O across the modified muskeg composites for 5 and 10 g of added mulch suggesting uniform infiltration of the silica precursor, conformal coverage, and homogeneous distribution of the components of the modified muskeg composite (Supplementary Figure 5).

**Figure 4:**
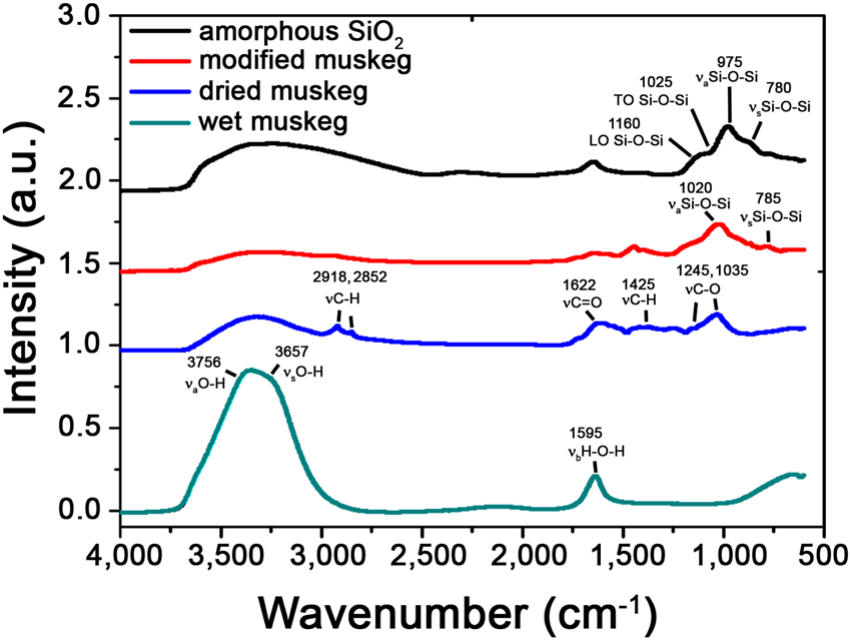
Infrared Spectroscopy of Muskeg and Modified Composites. FTIR spectra of wet, dried, and modified muskeg, and an amorphous SiO_2_ control sample prepared by reacting only Na_2_SiO_3_ with NH_4_OH. The stabilization of a siloxane –Si-O-Si- framework and the substantial reduction of water content is clearly established.

The nature of the silica matrix has been examined by Fourier transform infrared (FTIR) spectroscopy of the modified muskeg composites as depicted in Fig. 4. The as-prepared wet muskeg mainly shows strong characteristic vibrations of water, which is unsurprising given the high moisture content. Specifically, the symmetric stretching (3657 cm^−1^), asymmetric stretching (3756 cm^−1^), and bending (1595 cm^−1^) modes of water are discernible^32,33^. After drying of the muskeg under ambient conditions, infrared-active modes characteristic of organic matter are observed as assigned in Fig. 4 ^34^. The modes at 3600‒3700 cm^−1^ and 1595 cm^−1^ are significantly diminished in relative intensity upon elimination of water. After modification of the initial muskeg soils with Na_2_SiO_3_, mulch, and hydroxyethylcellulose, the band at 3300‒3700 cm^−1^ is substantially diminished, attesting to the extrusion of trapped water from the modified composite matrices. In contrast, new IR bands are observed at 1000‒1500 cm^−1^. In order to facilitate the assignment of these bands, a control experiment has been performed wherein amorphous SiO_2_ has been prepared by reacting Na_2_SiO_3_ with NH_4_OH. The FTIR spectrum of the amorphous SiO_2_ sample thus prepared is also plotted with the specific mode assignments noted in Fig. 4. The observation of these modes for the modified muskeg unequivocally corroborate the stabilization of a siloxane framework.

### Modified Muskeg as a Load-Bearing Composite

**Figure 5:**
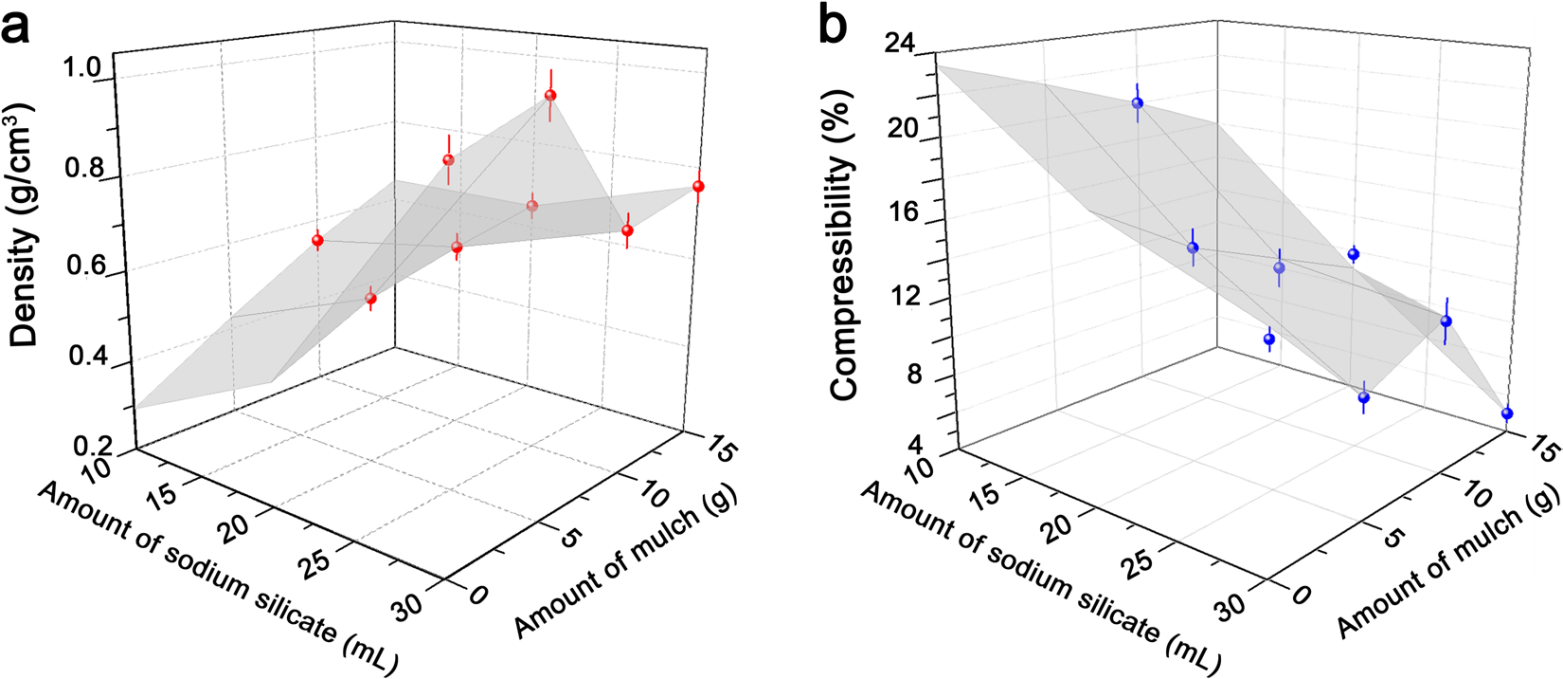
3D Mapping of Density and Compressibility of Modified Muskeg Composites as a Function of Added Precursors. (**a**) Density and (**b**) compressibility of modified muskeg as a function of the amount of added Na_2_SiO_3_ and mulch. The error bars depicted are calculated standard deviations for measurements made in triplicate.

The density of modified muskeg prepared using Na_2_SiO_3_ has been mapped as a function of the amount of added silicate precursor and mulch (Fig. 5a) since these two parameters most strongly influence the density values. Table 1 indicates that the amounts of added hydroxyethylcellulose and NH_4_OH appear to have less of an influence on the measured density; varying the amount of NH_4_OH from 5–15 mL while keeping the amount of Na_2_SiO_3_ and mulch constant at 20 mL and 10 g respectively, yields relatively closely spaced density values in the range of 0.62–0.70 g·cm^−3^. In contrast, the density of modified muskeg increases monotonically as a function of the amount of Na_2_SiO_3_ used from 0.55 g·cm^−3^ (10 mL), to 0.61 g·cm^−3^ (20 mL), and eventually to 0.72 g·cm^−3^ (30 mL) when the mulch content is held constant at 10 g (Table 1). Within this regime of compositional space, the addition of oligomeric silicate precursors gives rise to a denser siloxane matrix. These conditions also correspond to a homogeneous well-dispersed matrix, as discernible in Fig. 3b. The density of modified samples can be drastically increased up to 1.02 g·cm^−3^ by deploying 30 mL of Na_2_SiO_3_ and 5 g of mulch for the reinforcement of muskeg soils, which represents a dramatic improvement from the value of 0.20 g·cm^−3^ measured for unmodified muskeg. Given the relatively lower density of mulch (ca. 0.2 g·cm^−3^), inclusion of significantly greater amounts decreases the density. For a fixed amount of Na_2_SiO_3_ held constant at 30 mL, the addition of 15 g of mulch yields a density value of 0.76 g·cm^−3^,whereas the addition of 5 g of mulch leads to a density of 1.02 g·cm^−3^ (Table 1). Nitrogen adsorption/desorption data is plotted in Supplementary Figure 6 (between *P*/*P*_0_ values of 0.05 and 3); Type III isotherms are measured for both modified and unmodified muskeg samples with minimal interaction between nitrogen adsorbate molecules and the soil. The uptake of nitrogen is substantially reduced for the modified muskeg sample. A Brunauer—Emmett—Teller (BET) treatment of data suggests the specimen prepared using 30 mL Na_2_SiO_3_ and 15 g mulch has a surface area that is approximately halved from 3.41 m^2^·g^−1^ to 1.72 m^2^·g^−1^, further corroborating the measured densification (Supplementary Figure 6).

The mulch fibers are entangled to form a reinforcing network across the modified muskeg composite, whereas amorphous SiO_2_ is brittle, and thus mechanical properties, particularly compressibility, is not necessarily correlated with density. The increased strength of modified muskeg has been evaluated by measuring the compressibility as a function of applied weight. Supplementary Movie 1 illustrates that the cross-linked modified muskeg monoliths have a substantially modified appearance, mechanical resilience, and compressibility as compared to native muskeg. Supplementary Figure 7 depicts the placement of weights atop the modified muskeg; weights of up to 4 kg corresponding to pressures of ca. 20 kPa are applied and the height displacement is measured and plotted in Fig. 5b as a function of the amount of Na_2_SiO_3_ and mulch. The percentage compressibility as a function of added pressure is listed in Table 2.

**Table 2:** Compressibility of Modified Muskeg Composites. Measured compressibility for different modified muskeg compositions upon placement of solid weights as depicted in Supplementary Figure S7. Figure S7 shows representative examples for the acquisition of data shown here.

Sample Composition	Initial Displacement	5 kPa (1 kg)	10 kPa (2 kg)	20 kPa (4 kg)
Unmodified muskeg	0%	73.0 ± 8.1%	82.0 ± 10.5%	82.0 ± 10.5%
Na2SiO_3_ (30 mL)/Mulch (0 g)/NH_4_OH (10 mL)/Hydroxyethylcellulose (2 g)	0%	0%	7.1 ± 1.5%	14.5 ± 3.1%
Na2SiO_3_ (30 mL)/Mulch (0 g)/NH_4_OH (10 mL)/Hydroxyethylcellulose (4 g)	0%	0%	10.0 ± 2.9%	20.0 ± 3.5%
Na_2_SiO_3_ (20 mL)/Mulch (5 g)/NH_4_OH (10 mL)/Hydroxyethylcellulose (2 g)	0%	0%	8.0 ± 1.0%	15.0 ± 1.8%
Na_2_SiO_3_ (30 mL)/Mulch (5 g)/NH_4_OH (10 mL)/Hydroxyethylcellulose (2 g)	0%	0%	5.0 ± 0.7%	10.0 ± 1.5%
Na_2_SiO_3_ (30 mL)/Mulch (5 g)/NH_4_OH (10 mL)/Hydroxyethylcellulose (4 g)	0%	0%	6.3 ± 1.1%	12.5 ± 2.3%
Na_2_SiO_3_ (10 mL)/Mulch (10 g)/NH_4_OH (10 mL)/Hydroxyethylcellulose (2 g)	0%	0%	6.7 ± 0.7%	20.0 ± 2.1%
Na_2_SiO_3_ (20 mL)/Mulch (10 g)/NH_4_OH (10 mL)/Hydroxyethylcellulose (2 g)	0%	0%	6.3 ± 0.6%	12.5 ± 1.2%
Na_2_SiO_3_ (30 mL)/Mulch (10 g)/NH_4_OH (10 mL)/Hydroxyethylcellulose (2 g)	0%	0%	5.9 ± 1.1%	11.8 ± 2.3%
Na_2_SiO_3_ (20 mL)/Mulch (10 g)/NH_4_OH (5 mL)/Hydroxyethylcellulose (2 g)	0%	0%	8.3 ± 1.0%	16.7 ± 2.2%
Na_2_SiO_3_ (20 mL)/Mulch (10 g)/NH_4_OH (15 mL)/Hydroxyethylcellulose (2 g)	0%	0%	6.3 ± 0.5%	12.5 ± 1.0%
Na_2_SiO_3_ (20 mL)/Mulch (10 g)/NH_4_OH (10 mL)/Hydroxyethylcellulose (1 g)	0%	8.0 ± 0.7%	15.4 ± 1.8%	23.1 ± 2.9%
Na_2_SiO_3_ (20 mL)/Mulch (10 g)/NH_4_OH (10 mL)/Hydroxyethylcellulose (3 g)	0%	0%	7.7 ± 0.9%	15.4 ± 1.8%
Na_2_SiO_3_ (20 mL)/Mulch (15 g)/NH_4_OH (10 mL)/Hydroxyethylcellulose (2 g)	0%	0%	5.9 ± 0.5%	11.8 ± 1.0%
Na_2_SiO_3_ (30 mL)/Mulch (15 g)/NH_4_OH (10 mL)/Hydroxyethylcellulose (2 g)	0%	0%	2.5 ± 0.2%	5.0 ± 0.4%
Na_2_SiO_3_ (30 mL)/Mulch (15 g)/NH4OH (10 mL)/Hydroxyethylcellulose (4 g)	0%	0%	4.5 ± 0.3%	4.5 ± 0.3%

**Figure 6:**
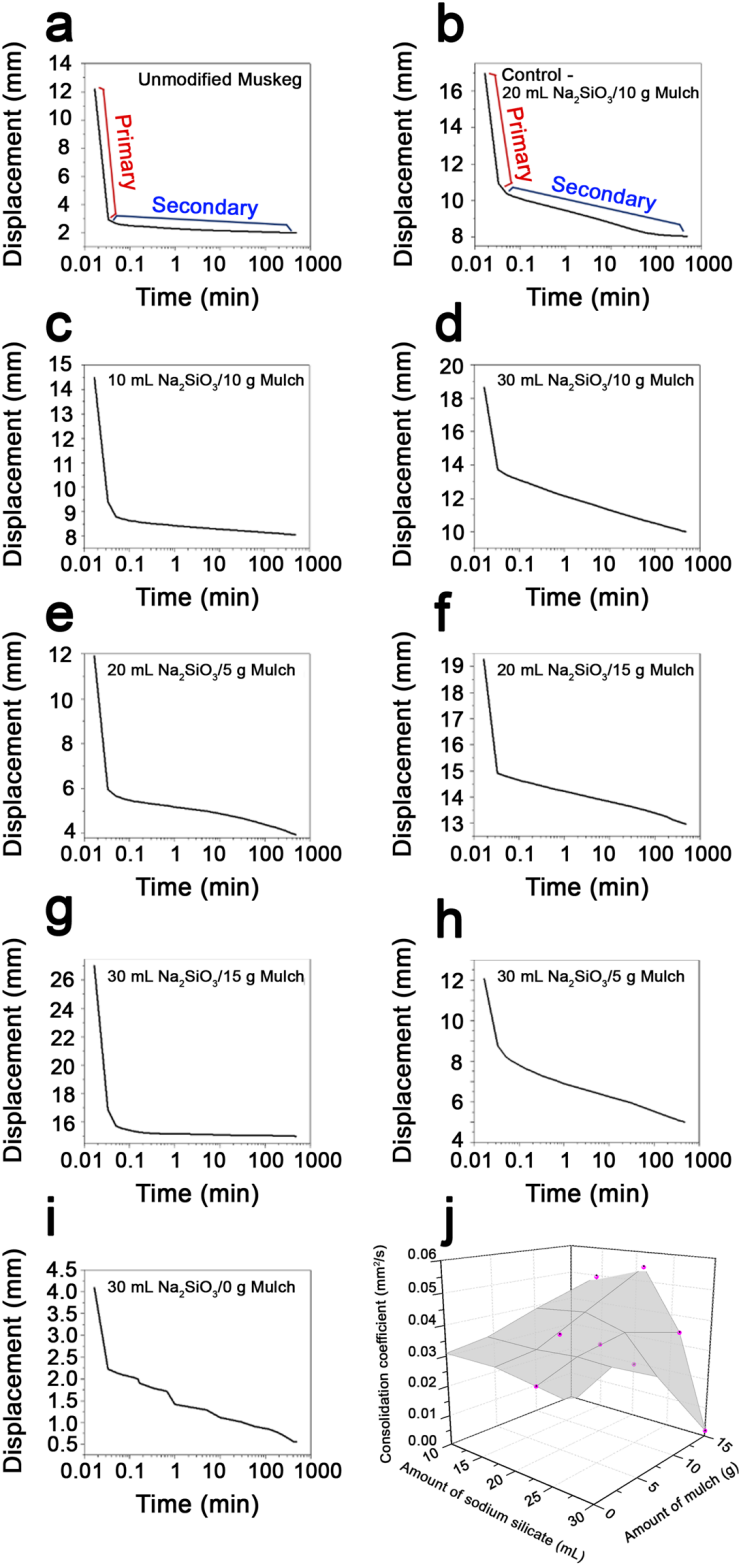
Consolidation Testing of Modified Muskeg Composites. Unmodified muskeg (**a**) and modified samples (**b**-**i**) containing varying amounts of Na_2_SiO_3_ (in mL) and mulch (in units of grams). The amount of NH_4_OH and hydroxyethylcellulose were held constant for each modified muskeg specimen at 10 mL and 2 g, respectively. The regions outlined in plots (**a**) and (**b**) indicate the primary (red) and secondary (blue) consolidation regimes. (**j**) 3D plot of coefficient of consolidation as a function of the amount of added Na_2_SiO_3_ and mulch. The measurement errors for the consolidation coefficient have been calculated through error propagation rules for elementary operations and functions.

Unmodified muskeg is strongly compressed with increasing weight as a result of the extrusion of water; Supplementary Figure 7 and Table 2 depict a remarkable 82% decrease in the height of a muskeg specimen subjected to a 20 kPa pressure applied by placement of a solid weight. Figure 5b indicates that the compressibility of modified muskeg monoliths is dramatically reduced with increasing addition of Na_2_SiO_3_ and mulch, consistent with the stabilization of more extensively cross-linked and densified frameworks. A combination of 30 mL of Na_2_SiO_3_ and 15 g of mulch limits the compressibility to only 4%, more than an order of magnitude improvement over unmodified muskeg. The 3D maps of density and compressibility plotted in Fig. 6 identify a useful compositional space wherein both parameters can be substantially enhanced as compared to unmodified muskeg.

Figure 6 and Table 3 present results of consolidation testing of muskeg as a function of the amounts of added Na_2_SiO_3_ and mulch. The amounts of hydroxyethylcellulose and NH_4_OH have been held constant at 2 g and 10 mL, respectively, based on optimal conditions identified from Tables 1 and 2. The consolidation tests performed here mimic the conditions of placement of a heavy load, either a road or a concrete slab, atop the modified muskeg. Because of the application of a heavy load (345 kPa over an 8 h time period), the initial compression normally observed upon preloading is absent and primary consolidation occurs rapidly within the first few seconds. During this stage, the excess pore water is extruded and the resulting void space is rapidly eliminated, as observed by a sharp drop in displacement^35–37^. Subsequently, the samples enter a secondary consolidation regime; the measured displacement in both regimes is plotted as a function of time on a logarithmic scale (Fig. 6). The initial and final heights, height percentage change, initial and final displacement, and change in displacement for each specimen examined in Fig. 6 are listed in Table 3. The time fitting method outlined by Lamb and Whitman has further been used to determine the coefficient of secondary consolidation for each sample^38^.

**Table 3:** Consolidation Testing of Modified Muskeg Composites. Measured height displacements and calculated coefficients of consolidation for modified muskeg composites for varying amounts of Na_2_SiO_3_ and mulch. All samples contained 10 mL of NH_4_OH and 2 g of hydroxyethylcellulose.

Sample composition	*H*_i_ (mm)	*H*_F_ (mm)	*H*%	*D*_i_ (mm)	*D*_F_ (mm)	Δ*D* (mm)	*C*_a_ (mm^2^/min)
Unmodified muskeg	12.2 ± 0.02	2.0 ± 0.01	83.6 ± 0.06%	9.268 ± 0.025	10.202 ± 0.025	0.934 ± 0.035	0.0085 ± 0.0002
Na_2_SiO_3_ (10 mL)/Mulch (10 g)	14.5 ± 0.04	8.0 ± 0.02	44.8 ± 0.25%	5.094 ± 0.025	6.446 ± 0.025	1.352 ± 0.035	0.0095 ± 0.0002
Na_2_SiO_3_ (20 mL)/Mulch (10 g)	19.3 ± 0.03	9.0 ± 0.02	53.4 ± 0.20%	8.364 ± 0.025	11.272 ± 0.025	2.908 ± 0.035	0.0310 ± 0.0001
Na_2_SiO_3_ (30 mL)/Mulch (10 g)	19.5 ± 0.04	10.0 ± 0.02	48.7 ± 0.31%	5.758 ± 0.025	9.490 ± 0.025	3.732 ± 0.035	0.0398 ± 0.0002
Na_2_SiO_3_ (20 mL)/Mulch (5 g)	13.0 ± 0.03	4.0 ± 0.01	69.2 ± 0.16%	7.032 ± 0.025	9.050 ± 0.025	2.018 ± 0.035	0.0381 ± 0.0002
Na_2_SiO_3_ (20 mL)/Mulch (10 g)	19.3 ± 0.04	9.0 ± 0.02	53.4 ± 0.16%	8.364 ± 0.025	11.272 ± 0.025	2.908 ± 0.035	0.0310 ± 0.0001
Na_2_SiO_3_ (20 mL)/Mulch (15 g)	23.2 ± 0.05	13.0 ± 0.03	43.9 ± 0.16%	8.280 ± 0.025	10.238 ± 0.025	1.958 ± 0.035	0.0194 ± 0.0001
Na_2_SiO_3_ (30 mL)/Mulch (0 g)	4.1 ± 0.01	0.5 ± 0.00	87.8 ± 0.05%	1.876 ± 0.025	3.544 ± 0.025	1.668 ± 0.035	0.0610 ± 0.0007
Na_2_SiO_3_ (30 mL)/Mulch (5 g)	12.0 ± 0.02	5.1 ± 0.01	57.5 ± 0.33%	3.306 ± 0.025	7.070 ± 0.025	3.764 ± 0.035	0.0613 ± 0.0003
Na_2_SiO_3_ (30 mL)/Mulch (10 g)	19.5 ± 0.04	10.0 ± 0.02	48.7 ± 0.31%	5.758 ± 0.025	9.490 ± 0.025	3.732 ± 0.035	0.0398 ± 0.0002
Na_2_SiO_3_ (30 mL)/Mulch (15 g)	27.0 ± 0.05	15.0 ± 0.03	44.4 ± 0.19%	10.142 ± 0.025	12.004 ± 0.025	1.862 ± 0.035	0.0018 ± 0.0001

Unmodified muskeg shows a dramatic 83.6% change in height, almost entirely in the initial few seconds, corresponding principally to primary consolidation (Fig. 6a). The coefficient of secondary consolidation measured for unmodified muskeg is low at 0.0085, but considering the sample was almost completely compressed in the primary consolidation regime by the extrusion of the finite amount of water contained in this sample, this value does not provide a meaningful baseline for improvement upon modification. In stark contrast, the modified muskeg samples show substantially reduced overall consolidation (reflecting a 20–50% decrease of consolidation as a result of incorporating the siloxane framework and mulch). The modified sample containing 20 mL Na_2_SiO_3_ and 15 g mulch (Fig. 6f) shows the lowest percentage height change, limited to 43.9% after a period of 8 h; much of the consolidation is seen to occur in the secondary consolidation regime. Indeed, Fig. 6 indicates that increased Na_2_SiO_3_ content strongly modifies the consolidation behavior; as a result of the extrusion of water, primary consolidation is greatly reduced and secondary consolidation becomes the predominant mode with behavior reminiscent of clays yielding a predictable subsidence profile. Upon increasing the amount of the Na_2_SiO_3_ precursor from 10 to 20 to 30 mL while keeping the amount of mulch constant at 10 g, Table 3 indicates that the overall change coefficient of secondary consolidation is monotonically increased from 0.0095 to 0.0310, and finally to 0.0398 mm^2^ min^−1^. Given the brittle nature of SiO_2_, these results suggest that the relative mulch content strongly influences the extent of consolidation. The use of higher silicate precursor concentrations increases the density of the composite but yields a brittle material that is consolidated relatively rapidly and to a greater extent under the applied load. Holding the Na_2_SiO_3_ amount constant at 20 mL and successively increasing the mulch content from 5 to 10 to 15 g results in a monotonic decrease of the percentage change in height from 69.2% to 53.4%, and finally to 43.9%; analogously, the coefficient of secondary consolidation is decreased from 0.0381 to 0.0310, and finally to 0.0194 mm^2^·min^−1^ (Table 3). Figure 6j plots the variation of the coefficient of secondary consolidation as a function of the Na_2_SiO_3_ and mulch content. These results as well as the dataset where Na_2_SiO_3_ content is kept constant at 30 mL and the mulch content is varied both underscore the critical need to have a sufficient content of reinforcing mulch fibers without which the intrinsic brittle nature of amorphous silica ceramics, which are subject to fracture under compression is manifested. The percolative network of mulch fibers thus provides a mechanism for load transfer that is not otherwise accessible to the siloxane framework alone. The specimen containing 30 mL Na_2_SiO_3_ and 15 g mulch yields an attractive secondary consolidation coefficient value of 0.0018 mm^2^·min^−1^ and an overall change in height limited to ca. 44.4%.

**Figure 7:**
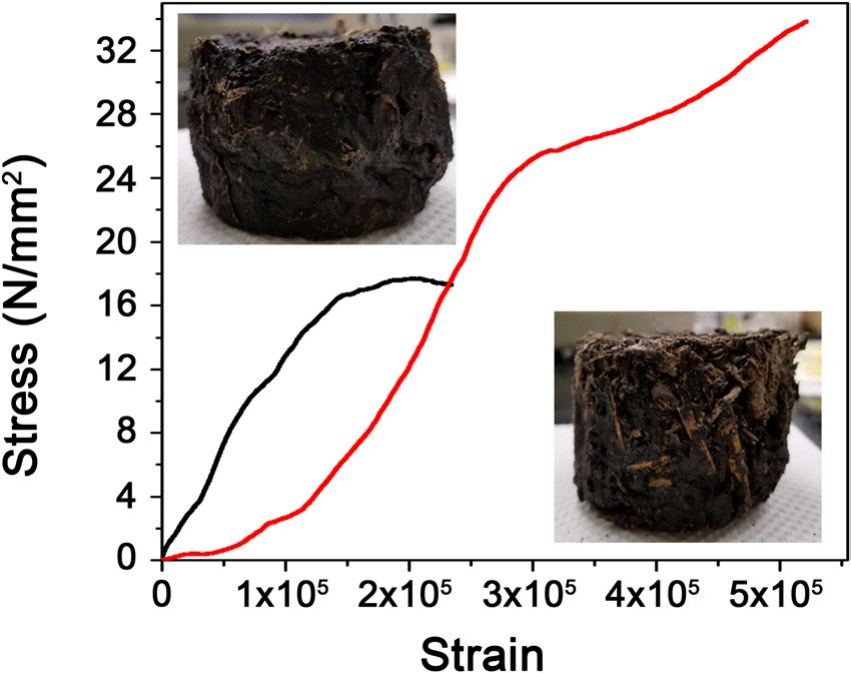
Compressive Testing of Muskeg Composites. Stress *versus* strain curves measured for modified muskeg samples containing 5 g of mulch (black) and 15 g of mulch (red) while keeping the remaining additives constant at 30 mL Na_2_SiO_3_, 10 mL NH_4_OH, and 2 g hydroxyethylcellulose. The insets depict photographs of the individual samples tested here.

Compressive testing has further been performed for the modified muskeg samples. While wet unmodified muskeg cannot be cast into an appropriate test structure, typical compressive strengths for fibrous sphagnum peat are in the range of 3.5–11 kPa^36^. Figure 7 indicates that a modified muskeg sample with 30 mL Na_2_SiO_3_ and 5 g mulch yields a compressive strength of 17.7 MPa; increasing the mulch content to 15 g increases the compressive strength to 33.8 MPa despite a reduction in density (Table 1). The observed enhancement in compressive strength further attests to the role of mulch in forming an interpenetrating network that is furthermore covalently bonded to the muskeg as a result of the formation of the siloxane framework (Fig. 1).

Recoverability of modified muskeg soil is paramount for reclamation and for environmental protection purposes. It is worth noting that several organosilanetriols has been designed recently that show improved biocompatibility, and can be further degraded through photocatalytic processes upon exposure to UV light^39^. Reclamation of the modified muskeg has been achieved through a digestion process with gypsum inducing the reverse condensation process (reverse reaction of Eq. 2) wherein dissolution of the silicate framework occurs upon treatment with a strong base (NaOH) and the sodium-ions are exchanged for calcium-ions from the gypsum. Elemental analysis shows the residual free sodium to be within the allowable limit of 200 mg·L^−1^ (specified by environmental regulatory agencies in Alberta) in the resulting mulch and muskeg. Future work will examine the inclusion of biodegradable matrices such as poly(ethylene glycol) in conjunction with photocatalytically degradable organosilanetriols^39^.

## Conclusions

Strengthening muskeg has been a formidable challenge with tremendous global implications for the fossil fuel industry given its increasing reliance on extraction of fossil fuel deposits from unconventional sub-Arctic deposits. The seeming intractability of muskeg arises from its high compressibility, high water content, and hollow cellular structure as well as the need to attain solidification in a reversible manner that preserves sub-Arctic habitats. In this work, we report a facile synthetic route for reinforcing muskeg with native mulch fibers with the help of inorganic silicate precursors that facilitate *in situ* formation of a siloxane network upon infiltration of liquid precursors between the pores of muskeg fibers. The approach developed here, based on catalyzed formation of a siloxane network with added cellulose providing a second cross-linking mode, allows for an increase of density as well as compressive strength while reducing the compressibility of the obtained load-bearing and low-subsidence composites.

## Methods

### Materials

The muskeg soils employed in this study were sourced from Cenovus Energy Inc.’s Foster Creek site in Alberta, Canada. Mulch, which is naturally occurring wood-fiber, was sourced from the same site. Tetraethylorthosilicate (TEOS; Strem chemicals) and Na_2_SiO_3_ (Spectrum chemical MFG Corp.), hydrochloric acid (HCl 36.5‒38.0%; Macron), ammonia solution (NH_4_OH 28‒30%; EMD Millipore), and hydroxyethylcellulose (Natrosol^TM^ 250H4Br PA; Ashland) were procured and used without further purification.

### Preparation of Densified Muskeg

A silicate matrix was constituted by infiltrating liquid-phase molecular or salt precursors within the muskeg followed by catalytic condensation^26,40,41^. Two different types of silica precursors, TEOS and Na_2_SiO_3_, were examined for the densification of muskeg soil. Initially, 10 g of wet muskeg soils were placed within a glass container. Next, fibrous frameworks (mulch), hydroxyethylcellulose, silicate precursors (either TEOS or Na_2_SiO_3_), and a condensation catalyst (either NH_4_OH or HCl) were added in varying amounts. The stoichiometric ratios of muskeg, mulch, silicate precursors, hydroxyethylcellulose, and catalyst were varied as depicted in Table 1. After mechanical blending of the mixtures, they were allowed to dry under ambient conditions, which typically required a period of 5‒7 days. The modified muskeg samples thus obtained were used for further characterization.

### Dissolution of Silicate Framework

The muskeg was arranged in a manner similar to topsoil conditions by placing 20 g of modified muskeg atop 20 g of unmodified muskeg within an open glass column, which was further plugged with glass wool at the collection end of the column. In order to simulate Alberta Agriculture and Forestry reclamation procedures within a laboratory environment, 3 cm of ground gypsum was then packed on top of the modified muskeg^42^. Subsequently, 250 mL of deionized water (Barnstead Nanopure system; *ρ* = 18.2 MΩ cm^−1^), 10^−1^ M NaOH, and 10^−4^ M NaOH were then used separately as three different pH modifiers to induce the hydrolysis of the siloxane linkages. The liquid was then collected and sent to AECOM Canada Ltd. for elemental analysis of free metals by Inductively Coupled Plasma – Mass Spectrometry (ICP-MS).

### Characterization

The pH value of muskeg soils was characterized by using a digital pH meter (HQ411d Benchtop pH/mV Meter) equipped with a glass electrode probe. A standard procedure for calibrating pH meters was implemented by plotting the measured potential as a function of pH. The morphological characteristics of the muskeg specimens before and after modification were evaluated using a Leica EZ4 stereomicroscope equipped with KL 1500 LCD and a FEI Quanta 600 field emission scanning electron microscope (FE-SEM) equipped with a conventional Everhart-Thornley detector, back-scattered electron detector, and IR-CCD chamber camera. An accelerating voltage of 10–20 kV was used to image the muskeg. The chemical composition of muskeg was evaluated by energy-dispersive X-ray spectroscopy (EDS) using an Oxford Instruments silicon drift detector. Fourier transform infrared (FT-IR) spectra were obtained using a Bruker VERTEX 70 instrument in the range of 4000–500 cm^−1^ with a spectral resolution of 4 cm^−1^.

### Calculation of density

The density of modified muskeg soils was calculated by placing 30 g of modified muskeg within a calibrated measuring cylinder and dividing the obtained final mass of the modified muskeg by the volume of the cylinder occupied. The density measurements were made in triplicate.

### Compressibility testing

The compressibility of the modified muskeg samples was evaluated by placing calibrated weights atop free-standing muskeg pucks with dimensions of 6.0 cm in diameter and 1.2–3.0 cm in height placed on a laboratory bench. The change in the height of the muskeg was measured in each instance using a 30 cm ruler at a measurement resolution of 1 mm. The height displacement (change in %) was calculated by dividing the height difference between initial and final by initial height.

4 $${\rm{Height}}\,{\rm{displacement}}\,( \% )=\frac{{H}_{i}\,-\,{H}_{f}}{{H}_{i}}\times 100 \% $$

where *H*_*i*_ is initial height and *H*_*f*_ is final height.

### Coefficient of consolidation testing

Consolidation tests were performed based on ASTM D2435. A Humboldt Conmatic Consolidation frame with a Minilogger and digital indicator were used to perform the consolidation tests. A vertical load of 345 kPa was applied for 8 h until the specimens reached the maximum load and were then unloaded. Coefficient of secondary consolidation data was then collected with the help of the Humboldt Materials Testing Software. The time fitting method was used in order to determine the coefficient of secondary consolidation for the various free-standing pucks as per^37^:

5 $${{\rm{C}}}_{{\rm{a}}}=\frac{\Delta {\rm{H}}/{{\rm{H}}}_{i}}{\Delta \,{\rm{log}}(t)}$$

where *H*_i_ is the initial height of the specimen, Δ*H* is the change in height over one log cycle, and *t* is the time in minutes.

### Testing of Compressive Strength

The compressive strength of the muskeg specimens were measured on an Instron 5982 Mechanical Testing Device using a servo-controlled 100 kN load capacity at a displacement rate of 5 mm per minute. The dimensions of the muskeg specimens measured 6.1 cm in diameter and 4.8 cm in height.

### Porosity measurements

Porosity measurements were performed using a Micromeritics ASAP 2420 Accelerated Surface Area and Porosimetry System at 77 K using ultra high purity (UHP) nitrogen. Samples were degassed at 433 K for 12 h under dynamic vacuum prior to dosing with nitrogen. Surface areas and pore volumes were calculated using the Microactive software from Micromeritics based on the Brunauer, Emmett, and Teller model (BET).

### Data availability

The data that support the findings of this study are available from the corresponding author, S.B., upon request.

## Electronic supplementary material

Videos S1

Supporting Information
