# Supplementary material for: Building on Sub-Arctic Soil: Geopolymerization of Muskeg to a Densified Load-Bearing Composite
Source: Sci Rep. 2017 Nov 7;7:14711. doi: 10.1038/s41598-017-15115-z (PMC5676679; doi:10.1038/s41598-017-15115-z)
Supplement: Supplementary file 2 — Supporting Information [file 41598_2017_15115_MOESM2_ESM.pdf]

Building on Subartic Soils: Geopolymerization of Muskeg to a Densified Load-Bearing Composite

Gregory R. Waetzig,◙[1,2] Junsang Cho, ◙[1,2] Max Lacroix,[3] and Sarbajit Banerjee[1,2]*

◙ These authors contributed equally to this work.

[1]Department of Chemistry, Texas A&M University, College Station TX 77843-3255

[2]Department of Materials Science & Engineering, Texas A&M University, College Station TX 77843; *E-mail: banerjee@chem.tamu.edu

[3]Cenovus Energy, Inc., 500 Centre St. S., Calgary, AB T2P 0M5, Canada; *Email: max.lacroix@cenovus.com

Condition	pH
Na_2SiO_3 + HCl	0.37
TEOS + HCl	2.13
Muskeg alone	6.82
TEOS + NH_4OH	8.40
Na_2SiO_3 + NH_4OH	10.94

Supplementary Table 1: pH Measurements. pH values measured for reaction mixtures when different catalysts are used to constitute the silica framework.

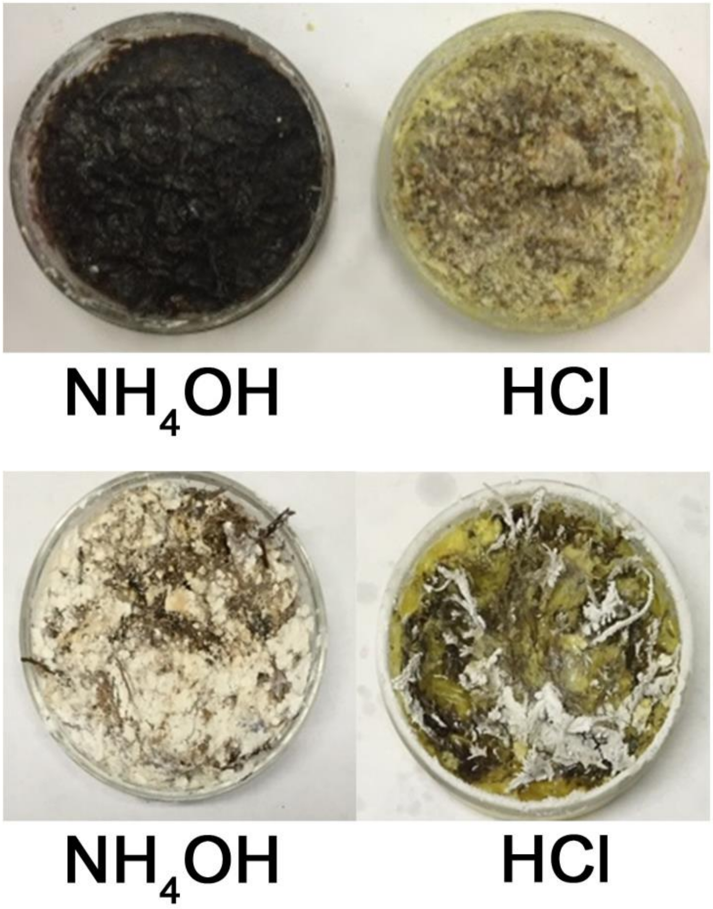

Supplementary Figure 1: Modified Muskeg at Different pH Conditions. Digital photographs of modified muskeg prepared using Na_2SiO_3 (top) or TEOS (bottom) with either NH_4OH (left) or HCl (right) as the catalyst. No mulch or hydroxyethylcellulose were used to prepare these samples. All samples depicted were prepared within a glass mold with a diameter of 6 cm.

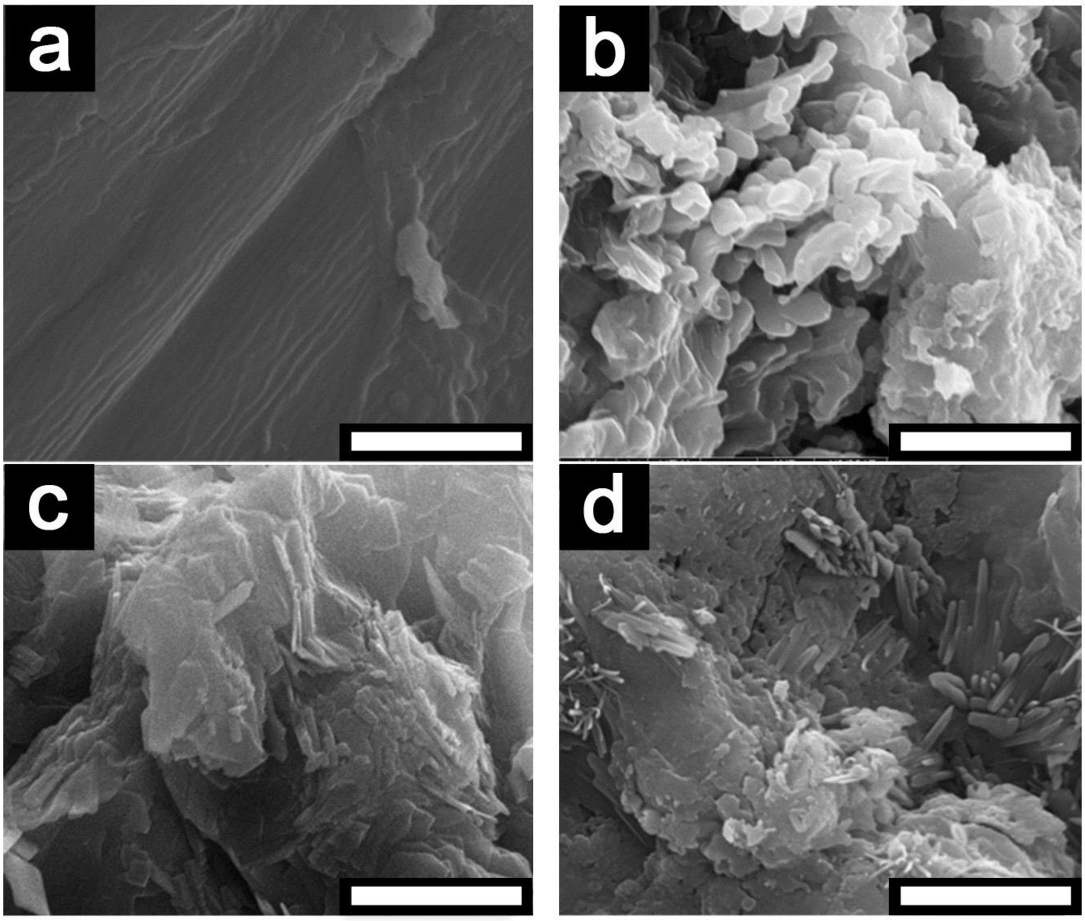

Supplementary Figure 2: Electron Microscopy Characterization of Modified Muskeg. Magnified SEM images of (a) unmodified muskeg, (b—d) modified muskeg with different ratios of Na_2SiO_3 and mulch; (b) 20 mL and 10 g, respectively; (c) 30 mL and 15 g, respectively; and (d) 30 mL and 5 g, respectively. The amount of NH_4OH and hydroxyethylcellulose were held constant for each modified muskeg specimen at 10 mL and 2 g, respectively. Scale bar = 4 µm.

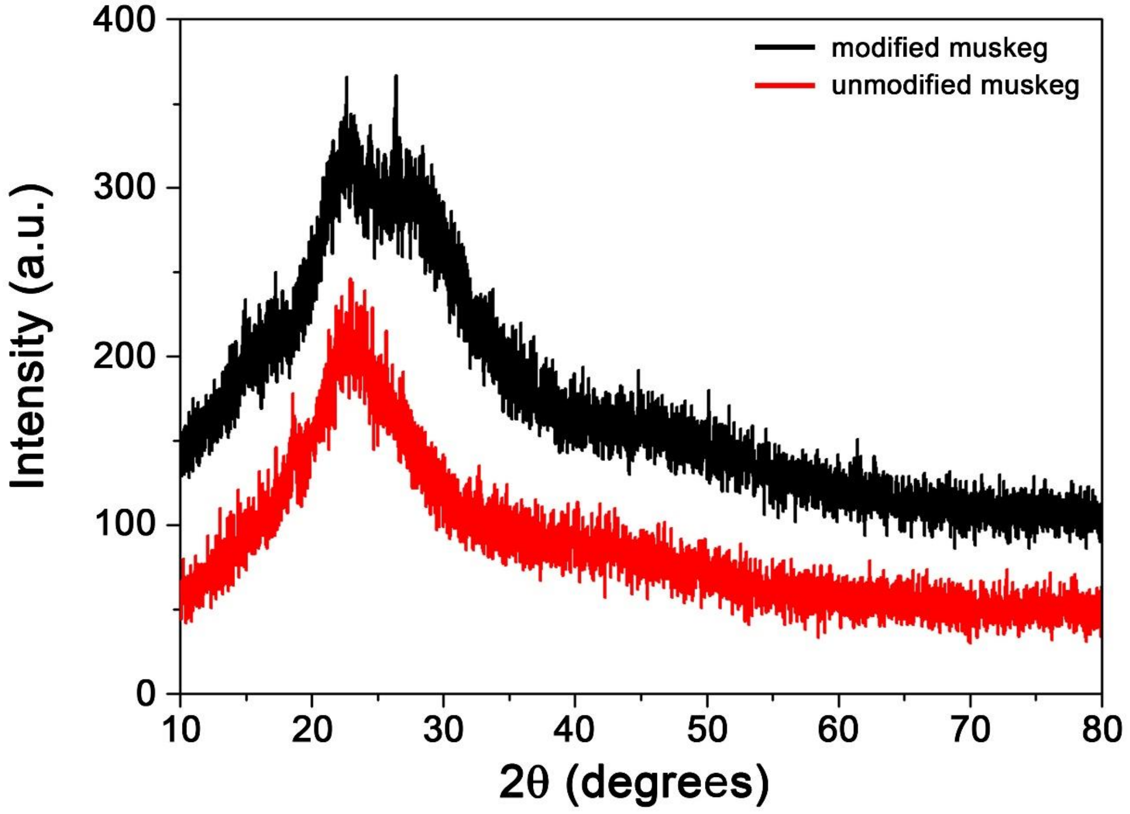

Supplementary Figure 3: X-ray Diffraction (XRD) Patterns of Modified Muskeg. Powder XRD pattern of unmodified muskeg (red) and modified muskeg (black) prepared using 20 mL Na_2SiO_3, 10 g mulch, 10 mL NH_4OH, and 2 g hydroxyethylcellulose. No evidence for crystalline species is discernible in the XRD patterns.

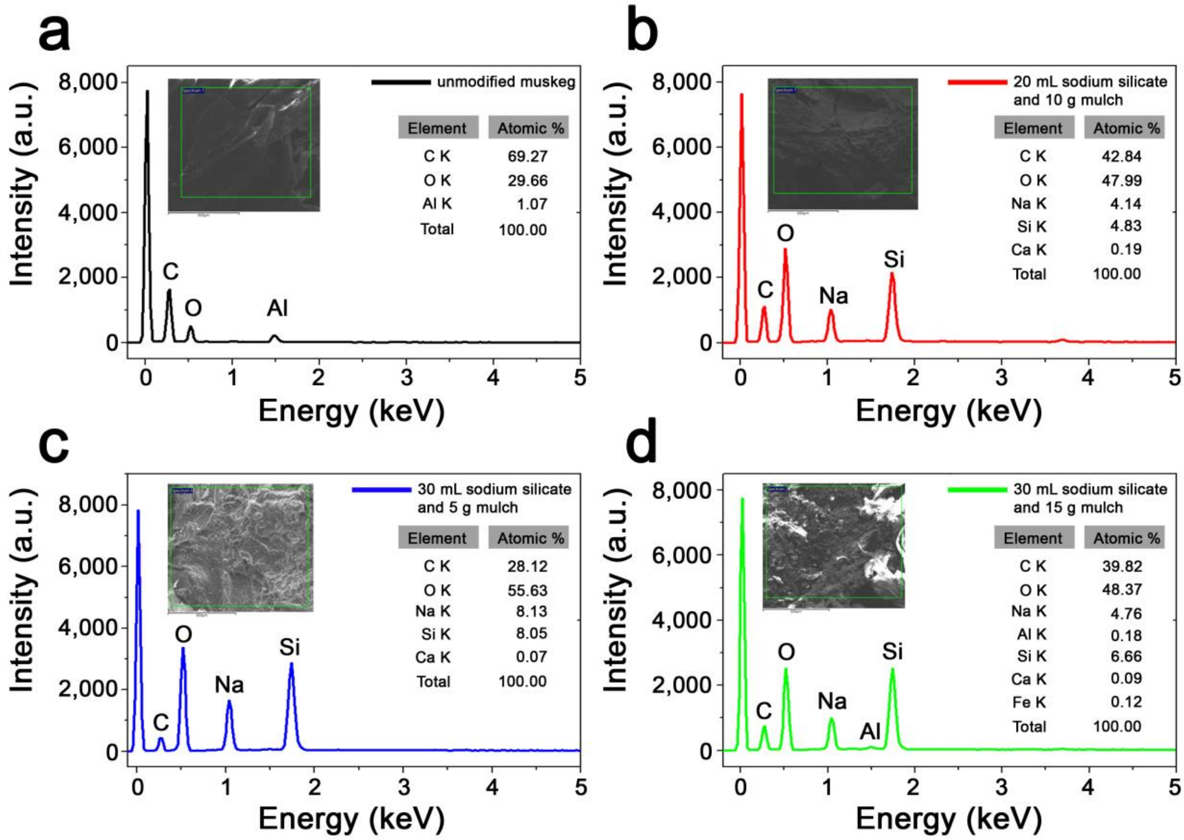

Supplementary Figure 4: Elemental Analysis of Muskeg Composites. EDS spectra of (a) unmodified and (b—d) modified muskeg with different added ratios of Na_2SiO_3 and mulch; (b) 20 mL and 10 g, respectively; (c) 30 mL and 5 g, respectively; and (d) 30 mL and 15 g, respectively. The insets illustrate SEM images of the areas examined by EDS. The amounts of NH_4OH and hydroxyethylcellulose were held constant for each modified muskeg specimen at 10 mL and 2 g, respectively.

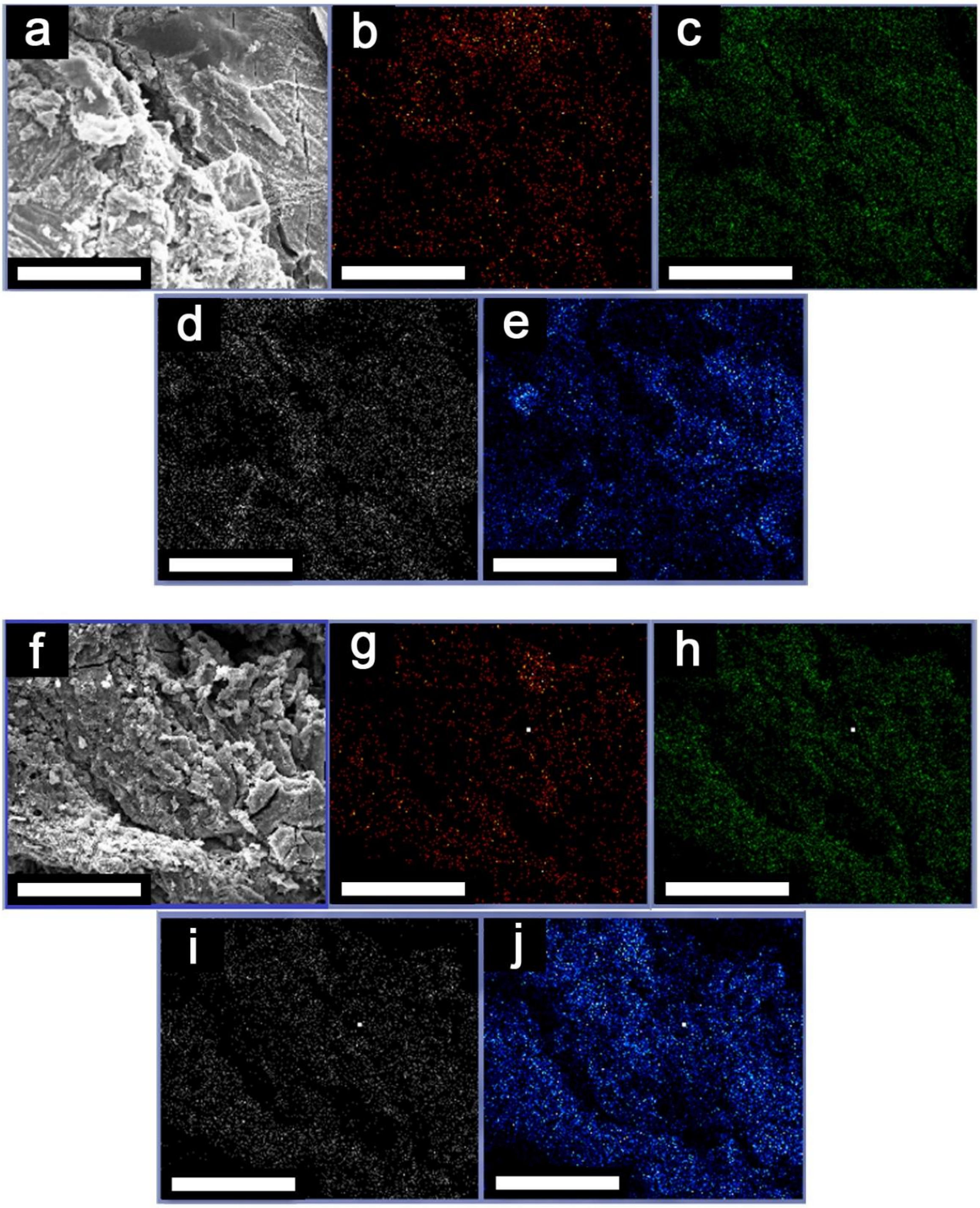

Supplementary Figure 5: Homogeneous Distribution of Components in Modified Muskeg Composites EDS color mapping of modified muskeg using (a—e) 20 mL Na_2SiO_3 and 10 g mulch and (f—j) 30 mL Na_2SiO_3 and 5 g mulch; (a,f) SEM images, (b, g) C K (red), (c,h) O K (green), (d, i) Na K (grey), (e,j) and Si K (blue). The amount of NH_4OH and hydroxyethylcellulose were held constant for each specimen at 10 mL and 2 g, respectively. Scale bar = 10 µm.

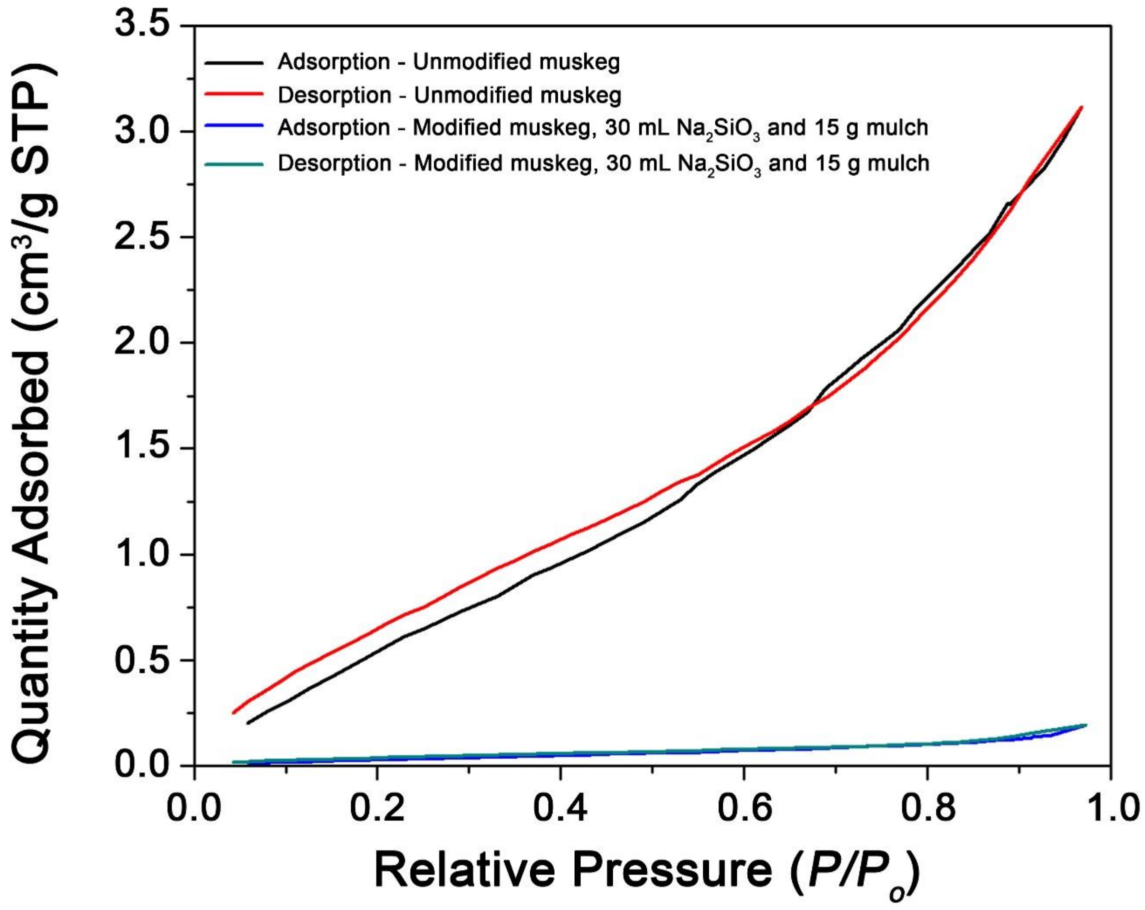

Supplementary Figure 6: Porosity Measurements of Muskeg Composites. Nitrogen adsorption/desorption plots measured for unmodified muskeg and modified muskeg laden with 30 mL Na_2SiO_3, 15 g mulch, 10 mL NH_4OH, and 2 g hydroxyethylcellulose.

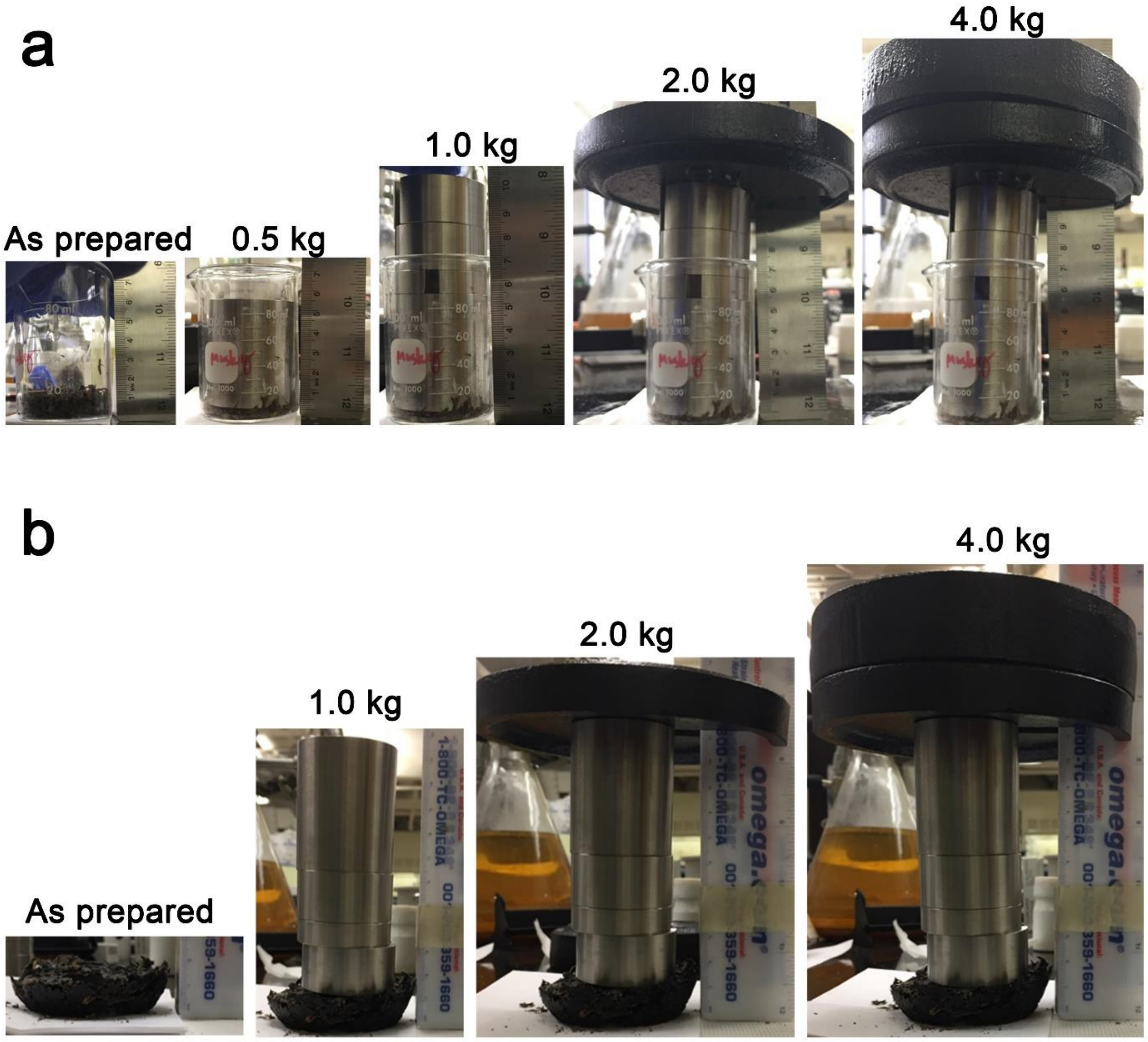

Supplementary Figure 7: Compressibility of Modified Muskeg Composites. Digital photographs of compressibility test for (a) unmodified muskeg (top) and (b) modified muskeg (bottom) as a function of weight placed on top of muskeg samples. The specific modified muskeg sample depicted here corresponds to 20 mL Na_2SiO_3, 10 g mulch, 10 mL of NH_4OH, and 2 g of hydroxyethylcellulose. See also **Supplementary Movie S1** that illustrates the contrast in compressibility for modified and unmodified muskeg.
